# Data on pro-inflammatory cytokines IL-1β, IL-17, and IL-6 in the peripheral blood of HIV-infected individuals

**DOI:** 10.1016/j.dib.2016.07.023

**Published:** 2016-07-19

**Authors:** Tommy Saing, Anddre Valdivia, Parveen Hussain, Judy Ly, Leslie Gonzalez, Frederick T Guilford, Daniel Pearce, Vishwanath Venketaraman

**Affiliations:** a Graduate College of Biomedical Sciences, Western University of Health Sciences, Pomona, CA 91766, USA; b Department of Basic Medical Sciences, College of Osteopathic Medicine of the Pacific, Western University of Health Sciences, Pomona, CA 91766-1854, USA; c Your Energy System, Palo Alto, CA, USA; d Riverside County Regional Medical Center, Moreno Valley, CA, USA

**Keywords:** Cytokines, HIV, Inflammation, Oxidative stress

## Abstract

Our most recent data indicate differences in the levels of pro-inflammatory cytokines (IL-1β, IL-17, and IL-6) and malondialdehyde (MDA), a stable end-product of lipid peroxidation in the plasma samples between HIV positive individuals with low CD4 T cell counts <200 mm^3^ and HIV positive individuals with CD4 T cell counts between 200 and 300 mm^3^ (ee). The data lend support and provide valuable correlation between CD4 T cell counts and the levels of inflammatory cytokines in HIV positive individuals.

## Specifications Table

**Table:** 

Subject area	*Biology*
More specific subject area	*Immunology, Infectious disease, oxidative stress*
Type of data	*Figures*
How data was acquired	*Data were obtained by using the Microplate Reader Instrument (Bio-Tek Multi-mode Instrument, VT, USA), KC4 Data Collection Software, and analyzed with Graph Pad Prism.*
Data format	*Raw and analyzed*
Experimental factors	*Whole blood from HIV-1 subjects with* CD4 T cell counts <200 mm^3^ [*n*=15] and CD4 T cell counts 200–300 mm^3^ will be referred as CD4>200} [*n*=15] *were obtained from the Riverside County Regional Medical Center in Moreno Valley, CA.*
Experimental features	*Whole blood specimen was processed using density gradient centrifugation in order to obtain plasma samples. ELISA and colorimetric kits were used for determining the levels of pro-inflammatory cytokines (IL-1β, IL-17, and IL-6) and MDA in the plasma obtained from individuals with HIV-1 infection*.
Data source location	*Department of Basic Medical Sciences, College of Osteopathic Medicine of the Pacific and Graduate College of Biological Sciences Western University of Health Sciences, Pomona, CA 91766.*
Data accessibility	*Data are in this article*

## Value of the data

• The data is important as it can provide researchers and medical practitioners a better understanding of the changes in the levels of pro-inflammatory cytokines and free radicals in HIV positive subjects with CD4 T cell counts <200 mm^3^ [*n*=15] and those with CD4 T cell counts >200 mm^3^ [*n*=15].
• The data shown in this article compares the levels of IL-1β, IL-17, IL-6 and MDA in the peripheral blood from HIV-1 positive individuals to the levels in healthy subjects [4], [5]. The findings may help understand the consequence of CD4 T cell decline in the pathophysiology of the disease process.
• Redox imbalance and exacerbated inflammation in HIV-1 positive individuals contribute to increased susceptibility for opportunistic infections [1], [2], [3]. These data could help researchers develop novel immunotherapeutics to modulate the host immune responses.

## 1 Data

**Fig. 1:**
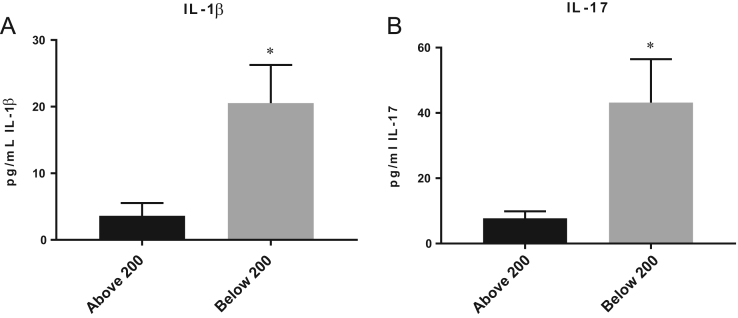
Levels of IL-1β and IL-17 in plasma from HIV-1 individuals with CD4 T cell counts >200 mm^3^ and CD4 T cell <200 mm^3^. Average values in normal healthy individuals range between 2.04±4.93 pg/mL [4]. Here we present values that show levels of IL-1β to be significantly higher in HIV-1 individuals with CD4 T cell <200 mm^3^ compared to CD4 T cell >200 mm^3^ (A). Data for IL-17 showed HIV-1 subjects with CD4 T cell <200 mm^3^ had significantly higher levels than subjects with CD4 T cell >200 mm^3^ (B). The average values found in peripheral blood of normal healthy individuals for IL-17 range between 6.53±7.42 pg/mL [4]. *P*-value ^*^p<0.05.

**Fig. 2:**
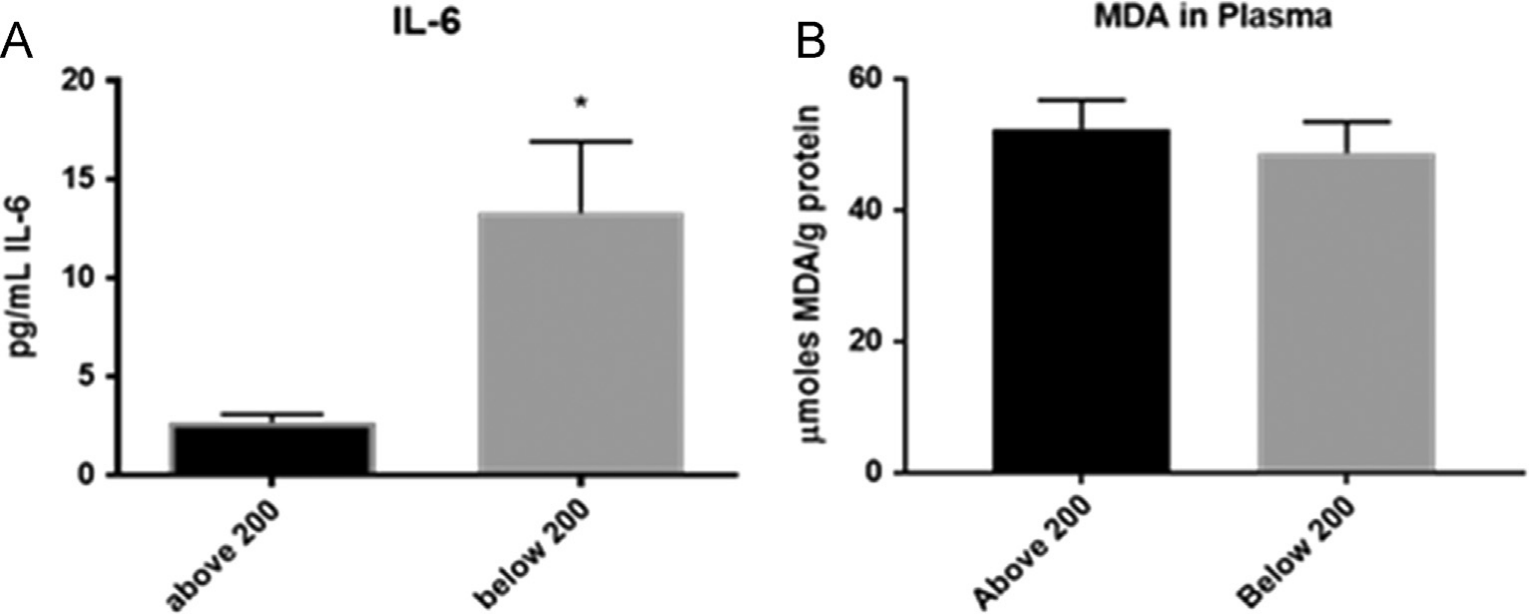
Levels of IL-6 and MDA in plasma samples from HIV-1 individuals with CD4 T cell >200 mm^3^ and CD4 T cell <200 mm^3^. HIV-1 subjects with CD4 T cell <200 mm^3^ had significantly higher levels of IL-6 than subjects with CD4 T cell >200 mm^3^ (A). Average values of IL-6 in healthy individuals range between 2.91±6.45 pg/mL [4]. Although it was not significant, levels of MDA were similar between HIV-1 subjects with CD4 T cell >200 mm^3^ and with CD4 T cell <200 mm^3^ (B). Levels of MDA from healthy subjects are significantly lower than HIV infected individuals [1], [2], [5]. *P*-value, ^*^p<0.05.

Our data indicates a correlation between diminished CD4 T cell counts and increased levels of pro-inflammatory cytokines (IL-1β, IL-17, and IL-6) that contribute to systemic inflammation and cell signaling [1], [2]. Our methodology for the measurement of these cytokines is colorimetric ELISA detection kits as previously reported [1], [2], [3]. The data that we presented here contribute to the understanding of the pathophysiology of the disease process in individuals with HIV-1 infection (Fig. 1, Fig. 2).

## 2 Experimental design, materials and methods

### 2.1 Study subjects and blood specimens collection

The Institutional Review Board of Western University of Health Sciences approved the research protocol. Blood specimens were obtained from HIV-1 positive participants recruited at the Riverside County Regional Medical Center, Moreno Valley, CA. Study subjects were under 65 years of ago with no preference for gender and ethnicity. Isolation of Plasma from Whole Blood.

Whole blood was collected from each subject and processed according to the method reported by our lab [1], [2]. Plasma samples were obtained from whole blood by performing density gradient centrifugation using Ficoll-Paque PLUS (10040757; GE Health care).

### 2.2 Malondialdehyde (MDA) measurement for oxidative stress

MDA is a byproduct of lipid peroxidation and is used to determine the levels of oxidative stress in the cells. A colorimetric is observed at 530–540 nm when MDA forms an adduct with thiobarbituric acid. Detailed protocol was previously reported [1], [2], [3].

### 2.3 Statistical analysis

Statistical data were analyzed using Graph Pad Prism Software. All data were reported in means *P*-values (*p*<0.05), using unpaired *t*-test with Welch׳s correction.

## References

[1] Saing T., Lagman M., Castrillon M., Gutierrez E., Guilford F., Venketaraman V. (2016). Analysis of glutathione levels in the brain tissue samples from HIV-1-positive individuals and subject with Alzheimer׳s disease and its implication in the pathophysiology of the disease process. BBA Clin..

[2] Ly J., Lagman M., Saing T., Singh M.K., Tudela E.V., Morris D., Anderson J., Davila J., Ochoa C., Patel N., Pearce D., Venketaraman V. (2015). Liposomal glutathione supplementation restores TH1 cytokine response to mycobacterium tuberculosis infection in HIV-infected individuals. J. Interferon Cytokine Res..

[3] Lagman M., Ly J., Saing T., Kaur Singh M., Vera Tudela E., Morris D., Chi P.T., Ochoa C., Sathananthan A., Venketaraman V. (2015). Investigating the causes for decreased levels of glutathione in individuals with type II diabetes. PLoS One.

[4] Kim H.O., Kim H.S., Youn J.C., Shin E.C., Park S. (2011). Serum cytokine profiles in healthy young and elderly population assessed using multiplexed bead-based immunoassays. J. Transl. Med..

[5] Morris D., Guerra C., Donohue C., Oh H., Khurasany M., Venketaraman V. (2012). Unveiling the mechanisms for decreased glutathione in individuals with HIV infection. Clin. Dev. Immunol..

