# Supplementary material for: Data on pro-inflammatory cytokines IL-1β, IL-17, and IL-6 in the peripheral blood of HIV-infected individuals
Source: Data Brief. 2016 Jul 19;8:1044–7. doi: 10.1016/j.dib.2016.07.023 (PMC4969237; doi:10.1016/j.dib.2016.07.023)
Supplement: Supplementary file 2 — Supplementary material [file mmc2.pptx]

## Slide 1
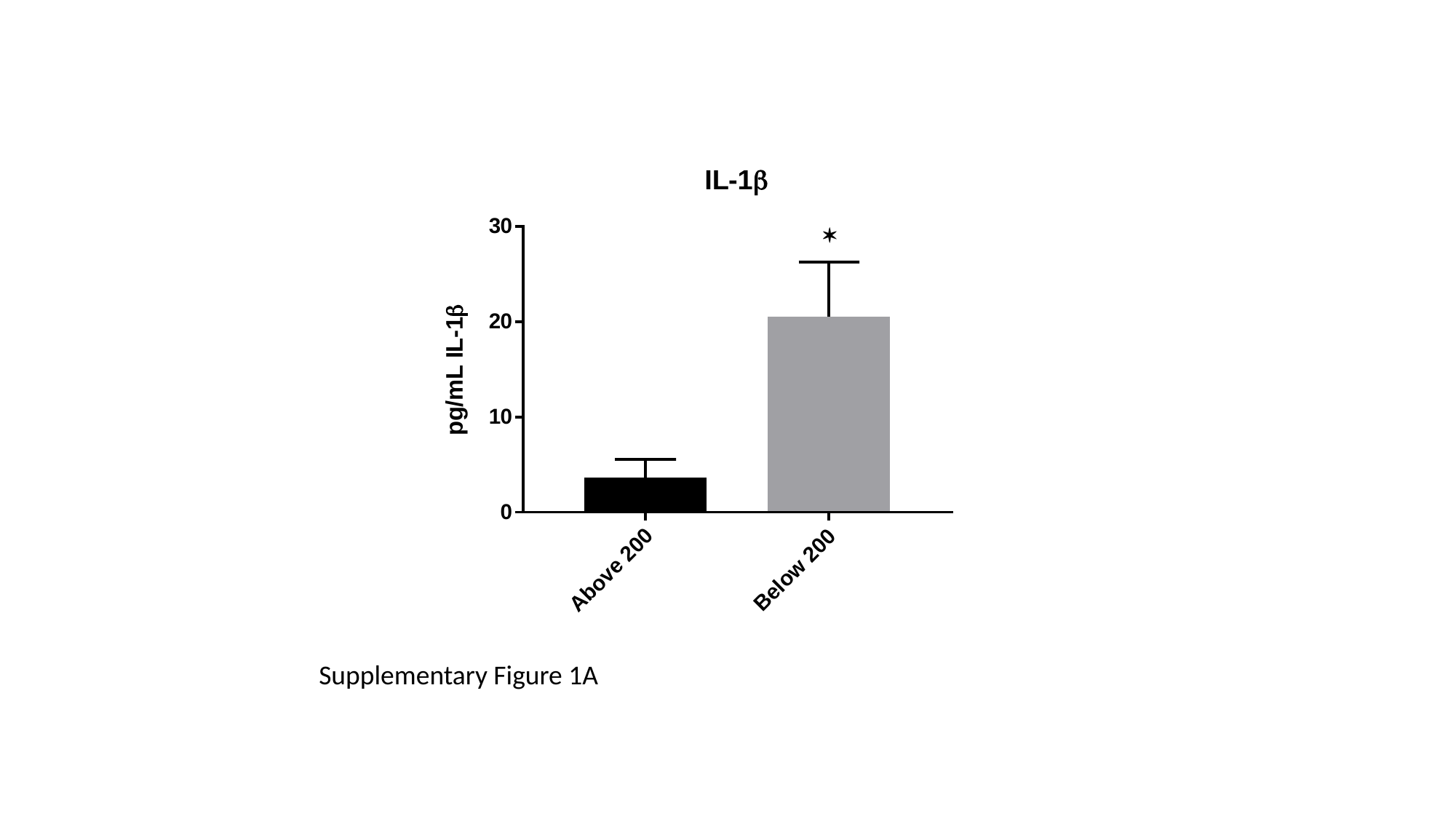

Supplementary Figure 1A

## Slide 2
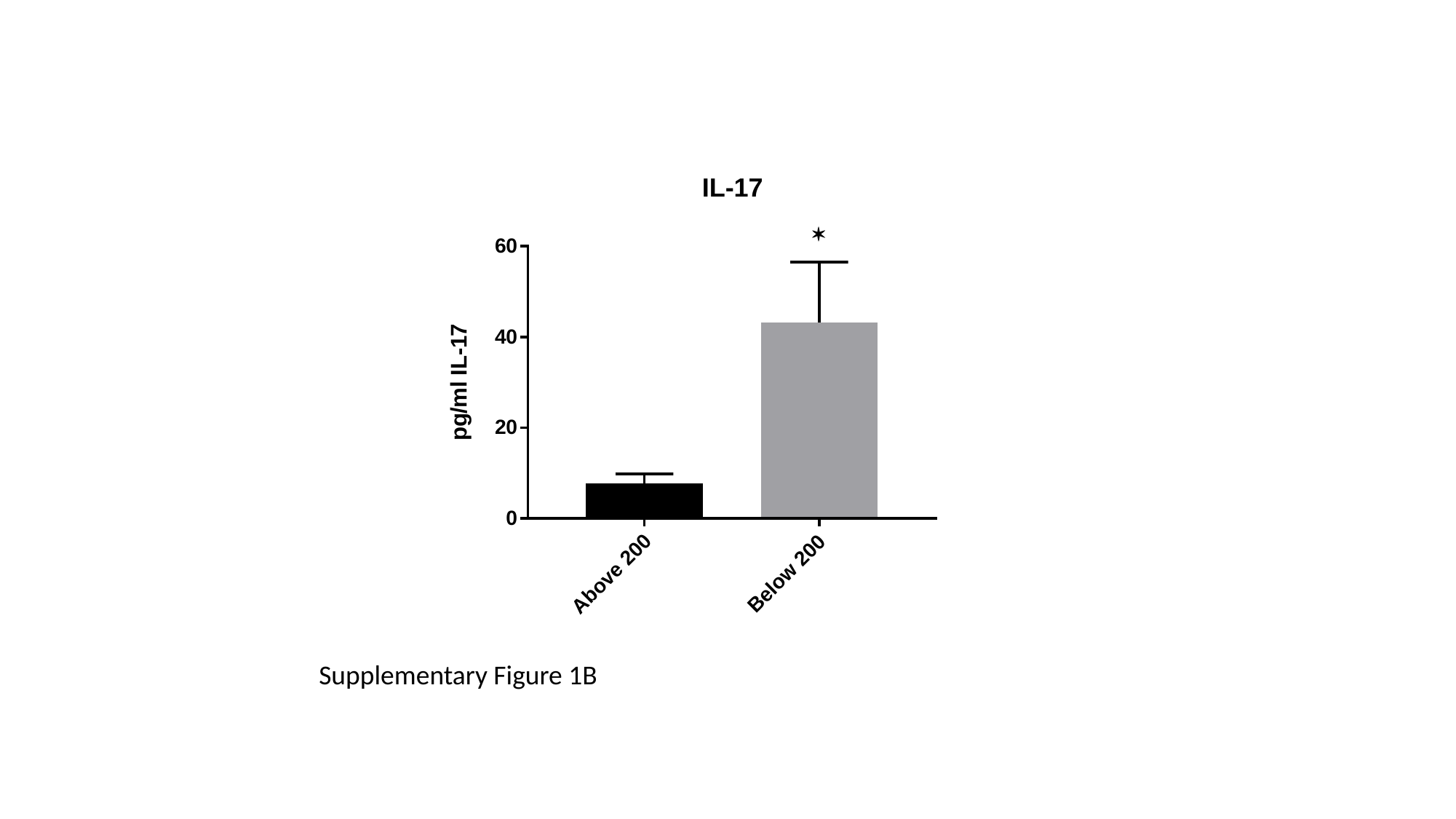

Supplementary Figure 1B

## Slide 3
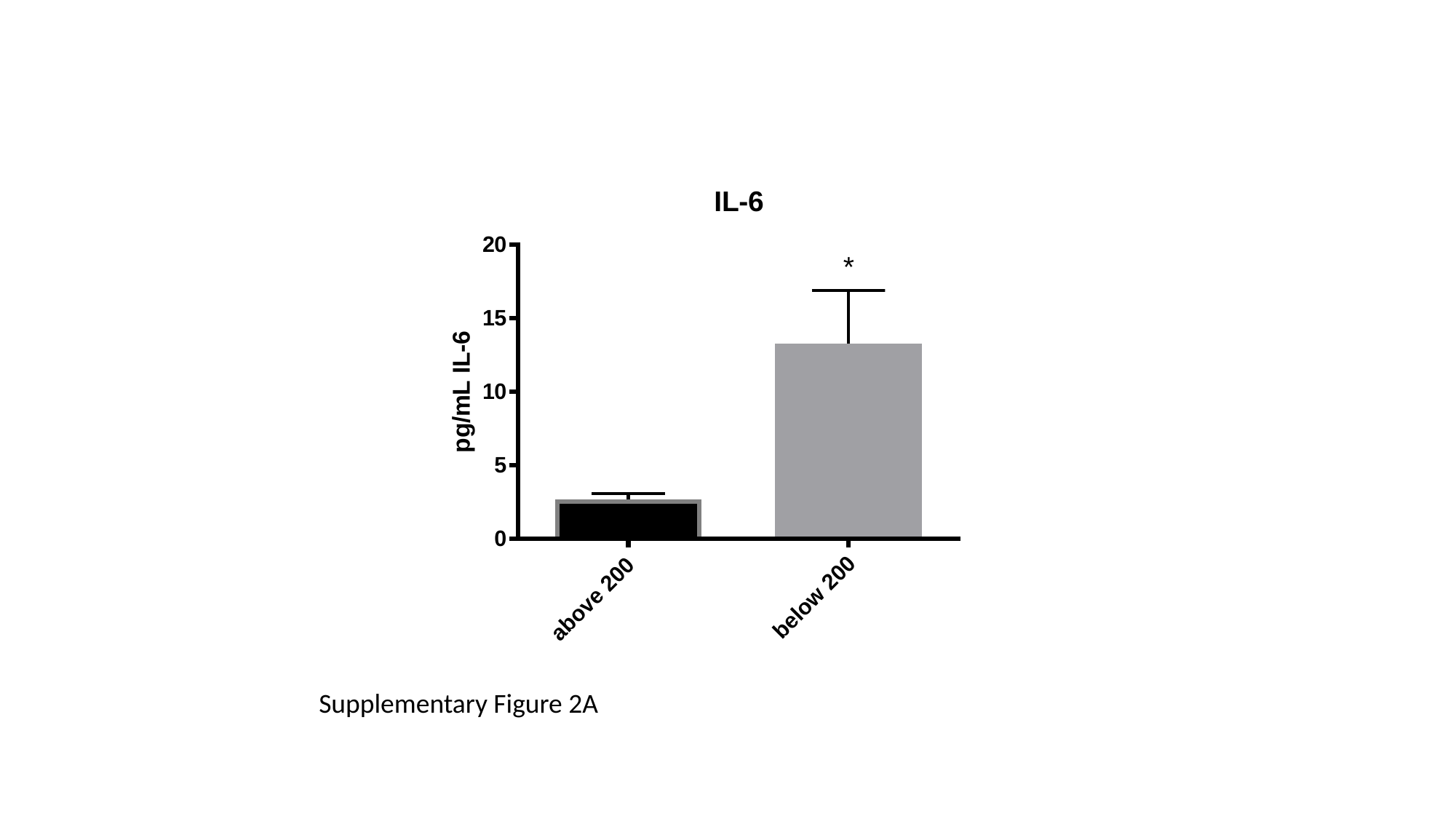

Supplementary Figure 2A

## Slide 4
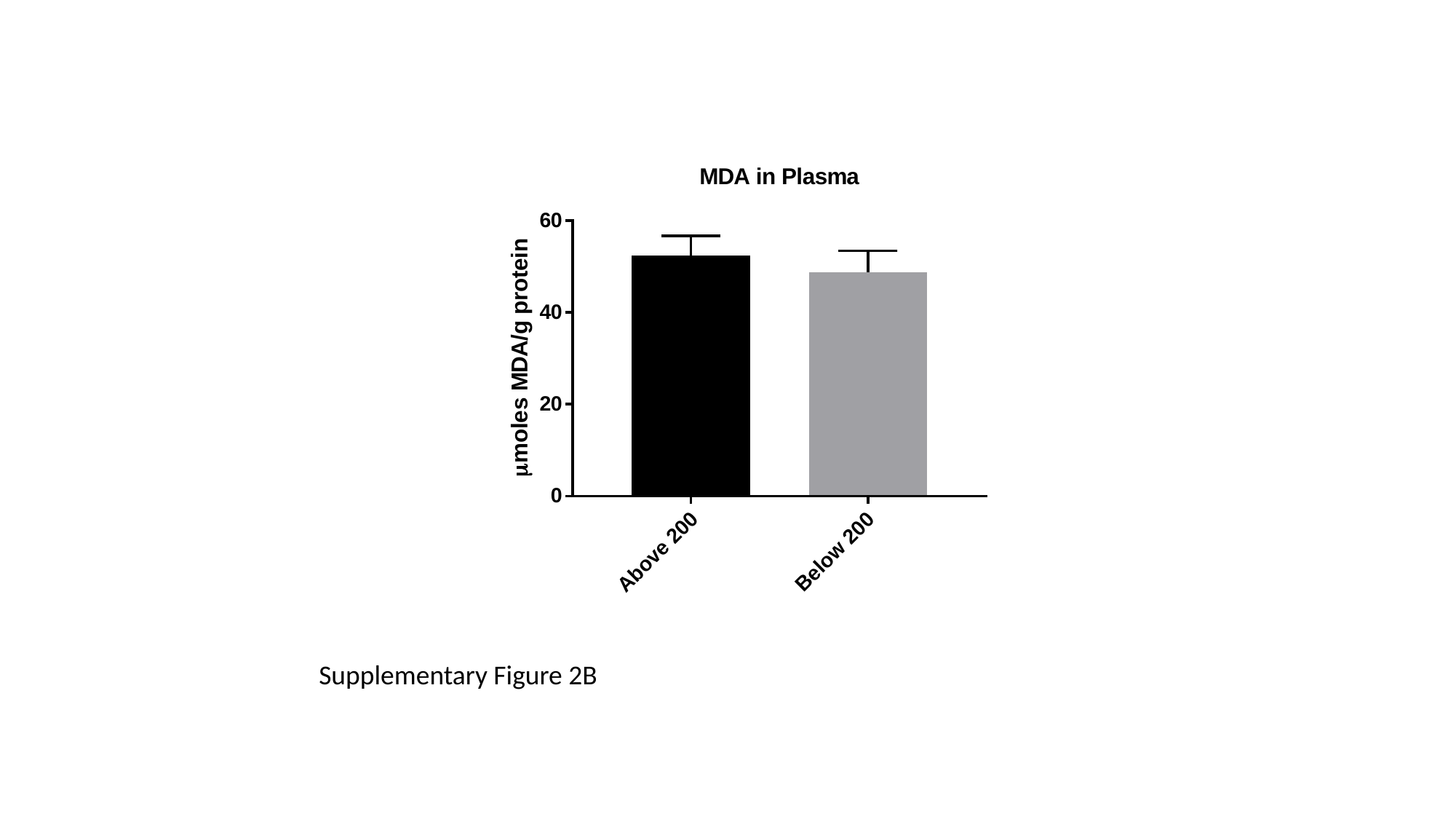

Supplementary Figure 2B
